# Biomaterial gels drive endogenous neurogenesis to support functional recovery after adult traumatic cortical injury

**DOI:** 10.4103/NRR.NRR-D-25-01291

**Published:** 2026-01-27

**Authors:** Jinting Wu, Peng Hao, Hongmei Duan, Wen Zhao, Yudan Gao, Zhiqiang Cui, Zhaoyang Yang, Xiaoguang Li

**Affiliations:** 1 Department of Neurosurgery, Tsinghua University Yuquan Hospital, School of Clinical Medicine, Tsinghua University, Beijing, China; 2 Department of Neurobiology, School of Basic Medical Sciences, Capital Medical University, Beijing, China

**Keywords:** biomaterial gel, chitosan–gelatin–collagen gel, endogenous neural stem cell, functional recovery, newborn neuron, traumatic cortical brain injury

## Abstract

Activation and neuronal differentiation of endogenous neural stem/progenitor cells within the hippocampal neurogenic niche (a neurogenic region) of the adult mammalian brain are considered potential bases for restoration of cognitive and memory functions. However, following traumatic cortical brain injury (i.e., injuries in a non-neurogenic region), the local microenvironment becomes hostile due to the presence of inhibitory factors such as inflammation, edema, and hypoxic–ischemic stress. These conditions hinder the migration and subsequent neuronal differentiation of activated neural stem/progenitor cells into the injured cortex. Numerous recent studies have confirmed that biomaterials can improve the microenvironment of the injured area and promote nerve regeneration. In this study, we report the findings obtained by injecting a biodegradable chitosan–gelatin–collagen gel into the cortical lesion area. We observed that the chitosan–gelatin–collagen gel established a permissive microenvironment for neural repair by suppressing inflammation and scar infiltration while promoting neovascularization. Thus, it enabled activated endogenous “seed” cells, i.e., neural stem/progenitor cells, to migrate into the cortical lesion area and generate new neurons and thereby facilitate functional recovery. These findings represent a novel biomaterial-based therapeutic strategy for the treatment of traumatic cortical brain injury, stroke, and other neurological disorders.

## Graphical abstract

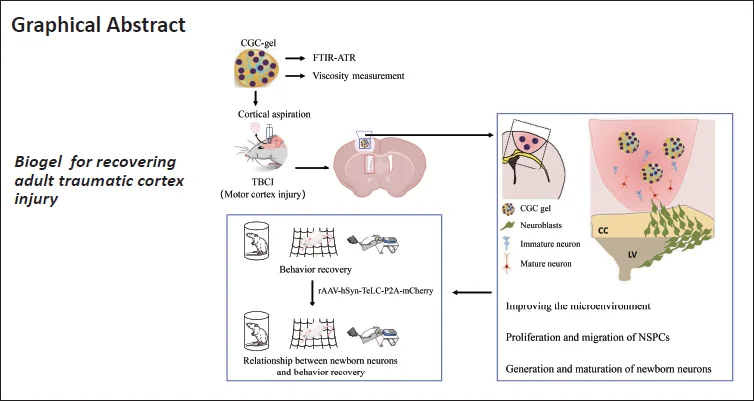

## Introduction

Although the adult mammalian central nervous system (CNS) retains a limited degree of plasticity, severe traumatic brain cortical injury (TBCI) usually causes irreversible loss of sensory, motor, autonomic, and cognitive functions (Truelle et al., 2010; Li et al., 2025). This factor has long supported the prevailing view that CNS neurons lack regenerative capacity. Following injury, the CNS microenvironment becomes profoundly hostile due to the presence of vascular rupture–induced ischemia and hypoxia, persistent and intense inflammatory responses, and glial scar infiltration (Truelle et al., 2010; Johnson et al., 2013; Muradashvili and Lominadze, 2013; Alves, 2014; Hinzman et al., 2016; Li et al., 2025). In addition to impeding neural regeneration and endogenous neurogenesis, these factors also tend to worsen over time, causing secondary injury and further restricting neuronal regeneration and functional recovery.

A growing body of evidence indicates that the adult CNS does harbor “seeds,” namely, neural stem/progenitor cells (NSPCs), for neural regeneration (Gage, 2000; Ohira, 2011). Under normal physiological conditions, NSPCs residing in the ependymal zone and subventricular zone (SVZ) surrounding the anterior lateral ventricles, as well as those in the subgranular zone of the hippocampal dentate gyrus continuously generate new neurons throughout life. These newborn neurons can integrate into existing neural circuits (Toni et al., 2008; Ihrie and Alvarez-Buylla, 2011). However, in the context of injury, inflammatory cytokines drive NSPCs toward astrocytic differentiation, contributing to glial scar formation. While glial scars can restrict the spread of inflammation (Zou et al., 2010), they cannot restore damaged neural circuits and instead create physical and chemical barriers that block axonal regeneration and elongation.

**Figure:**
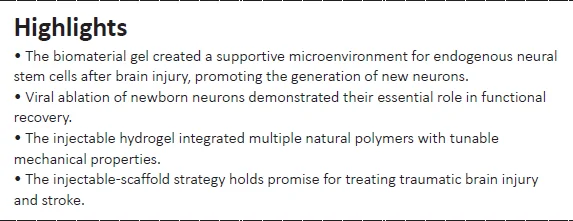

Even with the transplantation of exogenous neural stem cells (NSCs)—a potentially more robust regenerative source—neuronal replacement of lost host neurons requires a supportive microenvironment. Therefore, effective regulation of the injured area to establish an anti-inflammatory, neuroprotective (neurotrophic and pro-neurogenic) milieu is essential. Such an ideal microenvironment can not only preserve and protect residual neurons but can also promote the proliferation of endogenous (and/or exogenous) NSPCs and their differentiation into new neurons, thereby replacing lost neurons, enhancing neuroplasticity, and facilitating functional recovery.

In a previous study, we had employed a biodegradable chitosan–collagen scaffold to establish a robust anti-inflammatory environment that could reduce scar infiltration, facilitate the formation of new neural tissue after thoracic spinal cord injury, promote axonal regeneration, including that of the corticospinal tract, and induce the emergence of neuron-like cells, ultimately leading to the restoration of sensory and motor functions (Li et al., 2009). In the present study, we further modified our previously developed chitosan–collagen scaffold to create a chitosan–gelatin–collagen (CGC) gel (Li et al., 2009). This composite integrated the advantages of chitosan (a cationic polysaccharide), collagen (a negatively charged natural structural protein), and gelatin (a partially hydrolyzed derivative of collagen), providing excellent biocompatibility, tunable mechanical properties, and enhanced cell adhesion. These characteristics make the CGC gel well-suited for applications in tissue-engineering scaffolds, wound repair, and drug-delivery systems.

We injected the CGC gel into the cortical injury area of adult rats. Histopathological examination and behavioral testing revealed that the gel not only improved the local microenvironment (by suppressing inflammatory responses, reducing scar infiltration, and promoting vascular regeneration) but also induced robust neuroregeneration, enhanced neurogenesis, and facilitated functional recovery.

## Methods

### Animals

To eliminate the influence of estrogen’s protective effect on the brain, this study used adult male rats. Sixty adult Wistar rats (males; age: 8–10 weeks; weight: 220–250 g) were maintained three to a cage at room temperature with 60% relative humidity and a 12/12-hour dark/light cycle. The rats underwent focal aspiration cortical injury, as described previously (Li et al., 2025). All animal procedures were approved by the Animal Care and Use Committee of Capital Medical University (approval No. AEEI-2025-147; date: September 28, 2025) and complied with Beijing Laboratory Animal Association guidelines.

The animals were randomly divided into three groups, with 20 animals in each group: sham (rats underwent identical procedures without cortical tissue removal), lesion control (LC: rats received no graft after cortical removal), and treatment group (5 mg of CGC gel was implanted immediately into the cortical injured area after tissue removal).

### Preparation of the chitosan–gelatin–collagen gel

The CGC gel was generously provided by Beijing Zhishu Biotechnology Co., Ltd. Using a slightly modified version of the protocol from our previous study (Li et al., 2009), chitosan (degree of deacetylation ≥ 80%; molecular weight: 60–100 kDa) was dissolved in 0.5% acetic acid to prepare a 3% (w/v) chitosan solution. The pH was adjusted to 6.0–6.5, followed by filtration and sterilization. Collagen was dissolved in a neutral buffer at 4°C to a final concentration of 2 mg/mL. Gelatin powder was dissolved in deionized water in a 4060°C water bath to obtain a 10% (w/v) gelatin solution and stirred until fully clear.

The chitosan solution was mixed with the gelatin solution (kept at 40°C), and the collagen solution was gradually added at 4°C with gentle stirring to prevent local precipitation. The pH of the mixture was adjusted to 7.2–7.4 with NaOH or HCl to promote electrostatic crosslinking between the positively charged chitosan and negatively charged collagen/gelatin. The mixture was then incubated at 37°C to induce thermal gelation of collagen and gelatin, forming a network structure, and subsequently cooled to 4°C to enhance the physical crosslinking of chitosan. It was then centrifuged at 447 × *g* for 5 minutes at 4°C, after which the supernatant was discarded and the sample was stored at 4°C until use.

### Fourier transform infrared-attenuated total reflection spectroscopy

The CGC gel was analyzed using a Fourier transform infrared (FTIR)-attenuated total reflection (ATR) 7600 spectrometer (Lambda, Sydney, Australia) equipped with an ATR accessory. The scanning range was 400–4000 cm^–1^; the resolution was set to 2 cm^–1^; and 20 scans were averaged for each spectrum.

### Viscosity measurement

Viscosity was measured using an HR20 rheometer (TA Instruments, New Castle, DE, USA). Cold-stored samples were placed on a stainless-steel cone plate (diameter: 25 mm; cone angle: 2°), with the gap adjusted and trimmed. Measurements were performed at 37°C, with a shear strain of 0.1% and a shear rate of 1 Hz for 600 seconds. The ambient temperature and humidity during testing were 23°C and 40%, respectively.

### Traumatic brain cortical injury model and experimental groups

Briefly, the rats were intraperitoneally injected with 1 % pentobarbital sodium (50 mg/kg, Cat# Tc-P8411, Merck, Darmstadt, Germany) and fixed in a stereotaxic frame (RWD Life Science, China). Under sterile conditions, a craniotomy was made 2.8-4.8 mm posterior to bregma and 1–3 mm lateral to the midline on the left hemisphere. A custom-built tissue cutter (Chinese Patent No. 200810222343.7) was used to remove a cortical block (2 mm × 2 mm × 1.6 mm) without damaging the corpus callosum. The operation was performed using a previously described procedure (Li et al., 2025). The sham-operated rats underwent identical procedures without removal of cortical tissue. In the CGC group, 5 mg of CGC gel was implanted immediately into the cortical injured area after tissue removal. LC rats received no graft after cortical removal. All wounds were then sutured, disinfected, and treated with prophylactic antibiotics.

### Bromodeoxyuridine administration for labeling the fate of proliferated cells

To trace the fate of proliferated cells (Hao et al., 2017), all rats received intraperitoneal (IP) injections of bromodeoxyuridine (BrdU) (50 mg/kg in 0.9% saline; Sigma-Aldrich) immediately after brain injury, twice daily for 7 consecutive days after surgery.

### Multiplex immunofluorescence labeling for characterizing the effects of the chitosan–gelatin–collagen gel implantation on the injured brain microenvironment and endogenous neurogenesis

Four rats from each group were randomly chosen for multiplex immunofluorescence labeling. The rats were euthanized with 1% pentobarbital sodium (30 mg/kg, MilliporeSigma) injected intraperitoneally. After heart perfusion with 200 mL of normal saline followed by 400 mL of 4% paraformaldehyde (Aladdin, Shanghai, China), the brain was carefully removed, placed at 4°C, fixed with 4% paraformaldehyde for 1 day, and dehydrated for 1 day in 30% sugar phosphate buffer (PB). Next, the dehydrated brain tissue was placed in a –20°C cryostat for 30 minutes; embedded in optimal cutting temperature (OCT) compound (Sakura Tokyo, Japan); and sectioned into 30-μm continuous coronal slices using a cryostat microtome (CM1850 cryostat microtome, Leica, Wetzlar, Germany).

Briefly, serial coronal brain sections including the cortical lesion area were prepared using a cryostat and sequentially numbered. Either odd- or even-numbered sections were rinsed three times in phosphate-buffered saline (PBS), treated with 3% H_2_O_2_ for 10 minutes at room temperature to quench endogenous peroxidase activity, and then incubated for 1 hour at room temperature in a blocking solution containing 0.3% Triton X-100 and 10% normal goat serum (NGS). The sections were subsequently incubated overnight at 4°C with the primary antibodies.

For BrdU immunostaining, sections were pretreated in 2 N HCl at 37°C for 30 minutes to denature DNA, which was followed by neutralization in 0.1 M borate buffer (pH 8.4) at 37°C for 10 minutes. After blocking with NGS, the sections were rinsed in PBS and incubated for 1 hour at room temperature with biotinylated goat anti-mouse or anti-rabbit secondary antibodies (1:200; Vector Laboratories Inc., USA). This was followed by 1 hour of incubation with an avidin–biotin complex (ABC) kit (1:200; Vector Laboratories) and visualization using 5,5′-diaminobenzidine (DAB). Alternatively, after primary antibody incubation, the sections were incubated for 1 hour at room temperature with the appropriate fluorophore-conjugated secondary antibodies. Nuclei were counterstained with 4′,6-diamidino-2-phenylindole (DAPI; 1:3000; Sigma-Aldrich), and the sections were coverslipped. All primary and secondary antibodies used are listed in **Additional Table 1**.

**Additional Table 1:** Information regarding primary and secondary antibodies

Antibody	Source	Dilution	Catalog number	Supplier
Doublecortin (DCX, neuroblasts)	Rabbit	1:200	ab18723	Abcam, Cambridge, UK ZSGB Biotechnology, Beijing,
BrdU (proliferative cells)	Mouse	1:200	ZM-0013	China
Nestin (neural stem cells)	Rabbit	1:100	ab93157	Abcam
Tuj 1 (immature neurons)	Rabbit	1:500	T200020	Sigma, St. Louis, MO, USA
NeuN (mature neurons)	Rabbit	1:500	ab177487	Abcam
Glut-1 (vascular endothelial cells)	Rabbit	1:200	ab115730	Abcam
IBA1 (microglial cells)	Rabbit	1:500	ab178847	Abcam Molecular Probes, Eugene, OR,
Alexa Fluor 488 anti-mouse IgG	Goat	1:200	A-11001	USA
Alexa Fluor 488 anti-rabbit IgG	Goat	1:200	A-11008	Molecular Probes
Alexa Fluor 594 anti-rabbit IgG	Goat	1:200	A-11012	Molecular Probes
Alexa Fluor 647 anti-chicken IgY	Goat	1:200	A-21449	Molecular Probes

### Confocal imaging and quantitative analysis

#### Quantification of proliferated cells in the cortical lesion area

The number of BrdU-positive (BrdU^+^) cells was quantified using a confocal microscope (TCS SP8; Leica, Germany), as described previously (Hao et al., 2017; Li et al., 2025). Thirty serial sections (either odd- or even-numbered) including the cortical lesion area were randomly selected. Manual counts were performed for all BrdU^+^ nuclei within the lesion area, and only nuclei co-localized with DAPI staining were included in the analysis.

#### Quantification of double-labeled cells

Quantification of double-positive cells was conducted using established protocols (Bai et al., 2023). Briefly, double/triple immunofluorescence images were acquired in sequential scanning mode using a confocal microscope (TCS SP8; Leica, Germany). Ten coronal sections, each 30 μm thick, were collected at 180-μm intervals along the longitudinal axis of the lesion. Under high-power magnification, each BrdU⁺ nucleus was examined throughout the Z-axis, and only those completely surrounded by cytoplasmic signals of the specific phenotypic marker were classified as double-labeled. Cell counts for each marker were performed using a 25 μm × 25 μm counting frame (Hao et al., 2017; Li et al., 2025).

### Behavioral testing

Behavioral assessments included the cylinder test, grid-walking test, and grip-strength test to evaluate changes in forelimb sensorimotor function under different intervention conditions following cortical injury.

Rats were acclimated to the laboratory for approximately 10 minutes while the experimenter remained outside the room. The grip-strength meter was placed on a stable, flat surface, and the rat-specific testing grid was connected to a digital force gauge and disinfected with 75% ethanol or an appropriate disinfectant. The meter was then set up, connected to the testing system, and calibrated (zeroed) before testing.

Before testing, the contralateral forepaw of each rat was secured with medical adhesive tape to prevent it from gripping the meter. During testing, the experimenter gently held the base of the rat’s tail. Once the rat grasped the metal grid with the test-side forepaw, the tail was slowly pulled backward until the grip was released, and the grip force was recorded. The pulling speed was carefully controlled to remain slow enough for the rat to resist the force.

The peak force (in grams) displayed on the screen upon release from the grid was recorded (Bai et al., 2023). Baseline grip strength was assessed preoperatively, after which motor cortex injury surgery was performed. Postoperative behavioral evaluations of right forepaw grip strength were conducted on days 7, 14, 28, 56, and 90. During each assessment, grip strength was measured three consecutive times per rat, with at least 1 minute of rest between trials.

### Relationship between newborn neurons and behavioral improvement revealed by viral ablation techniques

If behavioral function in experimental animals was improved by implantation of the CGC gel, was this improvement attributable to the generation of newborn neurons in the cortical injury area? To address this question, tetanus toxin light chain (TeLC) was injected into the cortical lesion area to ablate the synaptic function of newborn neurons, thereby allowing investigation of the relationship between their synaptic activity and behavioral recovery in rats.

On postoperative day 35, the rats were anesthetized with Equithesin (3 mL/kg, i.p.) and fixed in a stereotaxic frame (RWD Life Science, China). Under sterile conditions, a craniotomy was made 2.8–4.8 mm posterior to bregma and 1-3 mm lateral to the midline on the left hemisphere, and the injury area was exposed. rAAV-hSyn-TeLC-P2A-mCherry (8.2 × 10^12^ µg/mL; Brain VTA Technology Co., Ltd.) was injected into the cortical lesion area of rats in the CGC gel group, specifically targeting the synaptic activity of neurons within the cortical lesion area. rAAV-hSyn-mCherry, which only expresses mCherry, was used as the control virus.

Behavioral assessments included the cylinder test, grid-walking test, and grip-strength test to evaluate changes in forelimb sensorimotor function under different intervention conditions following cortical injury.

TeLC is a protease that selectively cleaves proteins involved in synaptic vesicle exocytosis within target neurons, thereby inhibiting their synaptic function. This approach can elucidate the relationship between newborn neurons in the cortical injury area and behavioral functional recovery (Liang et al., 2019; Mu et al., 2025).

### Statistical analysis

All data were presented as the mean ± standard error of the mean (SEM). Statistical analyses were performed using SPSS 23.0 software (SPSS, IBM, NY, USA). Normality was assessed with the Shapiro–Wilk test, and homogeneity of variance was examined using Levene’s test. The comparison of measurement data differences between the two groups was performed using Student’s *t*-test. Differences between groups were compared using one-way analysis of variance followed by Tukey’s *post hoc* test. Multiple statistical analysis comparisons were performed by one-way analysis of variance. Bonferroni *post hoc* test was used to assess data variance, and Dunnett’s T3 *post hoc* test for data of unequal variance. Two-way analysis of variance were utilized for behavioral statistical analyses and Sholl analyses. Details on the statistical tests used for each experiment are provided in the results and figure legends. *P* < 0.05 was considered statistically significant (Li et al., 2025; Mu et al., 2025).

## Results

### Fourier transform infrared-attenuated total reflectance spectroscopy of the chitosan–gelatin–collagen gel

The FTIR-ATR analysis of the CGC gel revealed two characteristic infrared absorption peaks: the O–H and N–H stretching vibration peaks at 3435 cm^–1^, and the amide I band (corresponding to the C=O stretching vibration in the amide bond) at 1641 cm^–1^ (**Additional Figure 1**).

### Viscosity measurement of the chitosan–gelatin–collagen gel

The viscosity of the CGC hydrogel was measured using an HR20 rheometer (TA Instruments, New Castlr, DE, USA) at 37°C. Shear strain was set to 0.1% and shear frequency to 1 Hz. Each sample was tested for 600 seconds to obtain the viscosity profile over time (**Additional Figure 2**).

### Chitosan–gelatin–collagen gel promoted generation of newborn neurons in the injured cortical area of rats

We established an adult rat cortical injury model using a self-developed modified “biological tissue slicer” (Li et al., 2025). A tissue block (2.0 mm × 2.0 mm × 1.6 mm) was excised from the left motor cortex, which corresponds to the right forelimb motor representation area, while carefully avoiding damage to the corpus callosum. Gross examination of the brain specimens obtained at various postoperative time points revealed that the cortical injury sites in both the LC and CGC groups were filled with regenerative tissue. The nature of this regenerative tissue—whether it was a fluid-filled cavity with surrounding scar tissue or newly formed neural tissue containing neurons and blood vessels—was subsequently validated through histopathological analysis.

#### Chitosan–gelatin–collagen gel promoted proliferation and migration of neural progenitor cells in the subventricular zone after traumatic brain cortical injury

**Figure 1:**
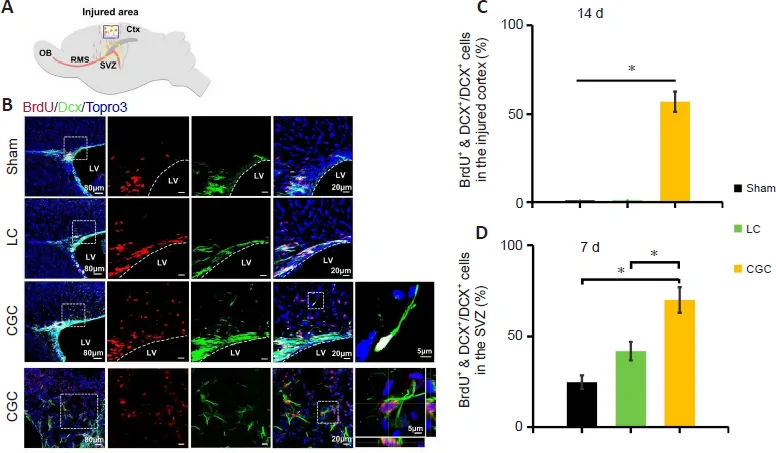
CGC promotes the proliferation and migration of neural progenitor cells in the SVZ following TBCI. (A) Schematic diagram illustrating the physical locations of the SVZ and cortical lesion area. (B) On day 7 after TBCI, BrdU^+^/DCX^+^ cells in the ipsilateral hemisphere of the Sham group were primarily confined to the SVZ, with no outward migration observed. In the LC group, the number of BrdU^+^/DCX^+^ cells in the ipsilateral SVZ was greater than that in the Sham group, with a few cells migrating away from the SVZ. In the CGC group, numerous BrdU^+^/DCX^+^ cells with elongated processes were scattered throughout the SVZ, and some BrdU^+^/DCX^+^ cells had migrated into the cortical lesion area (bottom row). Scale bars: 20 and 5 μm. (C, D) Quantitative analysis of the number of BrdU^+^/DCX^+^ neural progenitor cells in the cortical lesion and SVZ at different postoperative time points. Data are presented as the mean ± SEM (one-way analysis of variance followed by Tukey’s *post hoc* test). *P < 0.05. BrdU: Bromodeoxyuridine; CGC: chitosan–gelatin–collagen gel; DCX: doublecortin; LC: lesion control; SVZ: subventricular zone; TBCI: traumatic brain cortical injury.

Doublecortin (DCX) is a microtubule-associated protein commonly used as a marker of migrating neural precursor cells (Kempermann et al., 2018; Tobin et al., 2019; Berger et al., 2020). NSPCs retain proliferative activity during migration. To determine whether the proliferated cells (BrdU^+^ cells) observed in the cortical injury area originated from the proliferation and migration of stem/progenitor cells in the ipsilateral SVZ, BrdU/DCX double immunofluorescence labeling was performed on postoperative day 7 to assess the migratory status of SVZ NSPCs (**Figure 1A–D**).

On day 7 after TBCI, BrdU/DCX double-positive (BrdU^+^/DCX^+^) cells in the ipsilateral hemisphere of the Sham group were primarily confined to the SVZ and showed no outward migration. In the LC group, the number of BrdU^+^/DCX^+^ cells in the ipsilateral SVZ was greater than that in the sham control, with a small number of cells migrating away from the SVZ (*P* < 0.05; **Figure 1A**, **B**, and **D**). In the CGC group, numerous BrdU^+^/DCX^+^ cells with elongated processes were observed throughout the SVZ (**Figure 1B** and **D**). On day 14 after TBCI, BrdU^+^/DCX^+^ activated neural progenitor cells were observed within the cortical lesion area in the CGC group (*P* < 0.05; bottom row of **Figures 1B** and **3C**).

These findings suggest that following cortical injury, CGC gel implantation further recruited BrdU^+^/DCX^+^ cells from the SVZ and promoted their migration toward the cortical lesion area, where they exhibited prominent protrusions and branching. The morphological changes observed in these DCX-positive neural progenitor cells may provide a structural basis for brain repair.

#### Chitosan–gelatin–collagen gel promoted the generation and maturation of newborn neurons in the cortical lesion area after traumatic brain cortical injury

Under normal conditions, activated NSCs in the adult mammalian brain can differentiate into immature neurons, which subsequently mature into functional neurons (Duan et al., 2015; Yang et al., 2015). To investigate whether CGC facilitates the activation of endogenous NSPCs, their migration to the cortical lesion area, and their subsequent differentiation and maturation into neurons after TBCI, we examined the presence of BrdU/class III beta-tubulin (Tuj1) double-positive (BrdU^+^/Tuj1^+^) immature neurons in the cortical lesion area on postoperative day 21.

**Figure 2:**
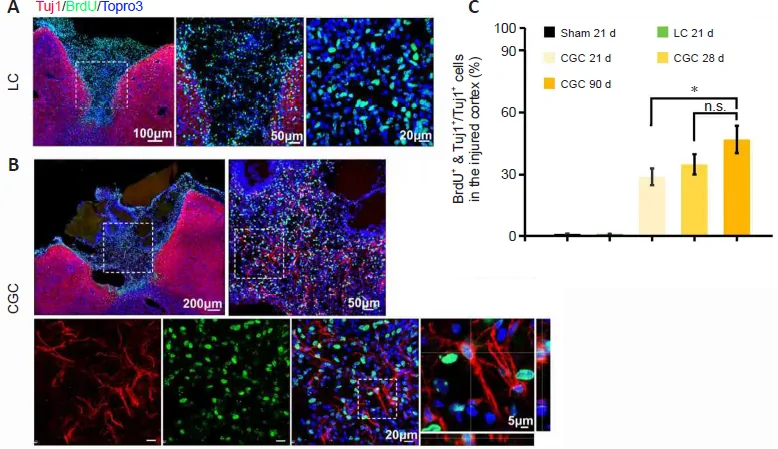
CGC promotes the generation of new immature neurons in the cortical lesion area after TBCI. (A) On postoperative day 21, no BrdU^+^/Tuj1^+^ newborn immature neurons were observed in the cortical lesion area of the LC group. Scale bars: 100, 50, and 20 μm. (B) In the CGC group, numerous BrdU^+^/Tuj1^+^ newborn immature neurons with pyramidal-like morphology were observed in the cortical lesion area. Scale bars: 200, 50, 20, and 5 μm. (C) Quantitative analysis of BrdU^+^/Tuj1^+^ newborn immature neurons in the cortical lesion area. Data are presented as the mean ± SEM (one-way analysis of variance followed by Tukey’s *post hoc* test). *P < 0.05. BrdU: Bromodeoxyuridine; CGC: chitosan–gelatin–collagen gel; LC: lesion control; n.s.: not significant; TBCI: traumatic brain cortical injury; Tuj1: class III beta-tubulin.

As shown in **Figure 2A–C**, no newborn Tuj1-positive (Tuj1^+^) cells (i.e., BrdU^+^/Tuj1^+^ cells) were detected in the lesion area of the LC group (**Figure 2A** and **C**). In contrast, the CGC group exhibited a substantial number of Tuj1^+^ cells, of which 29% co-expressed BrdU (**Figure 2C**). Some of these newborn neurons exhibited morphological features characteristic of cortical pyramidal neurons, including elongated and clearly branched processes. These findings suggest that the immature neurons (BrdU^+^/Tuj1^+^ cells) were generated within 7 days following TBCI. On postoperative day 28, the number of BrdU^+^/Tuj1^+^ cells further increased in the CGC group, and substantial numbers remained detectable in the cortical lesion area on postoperative day 90 (**Figure 2B** and **C**).

Neuronal nuclei (NeuN) is a marker of mature neurons (Gusel’nikova and Korzhevskiy, 2015; Yang et al., 2015). To further investigate whether the Tuj1^+^ immature neurons underwent maturation over time, BrdU/NeuN double immunostaining was performed on postoperative day 56 to identify mature neurons in the cortical lesion area.

**Figure 3:**
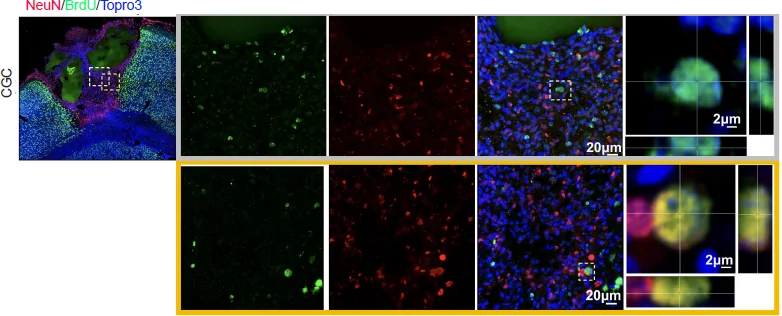
CGC promotes the generation of new mature neurons in the cortical lesion area after TBCI. On postoperative day 56, newborn BrdU^+^/NeuN^+^ mature neurons (within the yellow box) and BrdU^+^/NeuN^–^ cells were observed in the cortical injury area (top row). Scale bars: 20, 2 μm. BrdU: Bromodeoxyuridine; CGC: chitosan–gelatin–collagen gel; NeuN: neuronal nuclei; SVZ: subventricular zone.

In the LC group, almost no BrdU/NeuN double-positive (BrdU^+^/NeuN^+^) cells were detected in the cortical lesion area. In contrast, the CGC group exhibited NeuN-positive (NeuN^+^) cells, of which 21% were co-labeled with BrdU (**Figure 3**). These results indicate that CGC promoted the differentiation of NSCs into neurons and facilitated their maturation after TBCI.

### Role of the chitosan–gelatin–collagen gel in improving the cortical lesion microenvironment

The neuroprotective effects of CGC (including neurotrophic support and the promotion of neurogenesis) may be associated with its ability to create a favorable microenvironment in the cortical lesion area after TBCI. Accordingly, we investigated the influence of CGC on the microenvironment within the cortical injury area.

#### Chitosan–gelatin–collagen gel suppresses the inflammatory response following traumatic brain cortical injury

Inflammation and microglia play critical roles in the pathogenesis of TBCI (Hao et al., 2017). As the resident immune cells of the brain, microglia undergo morphological changes after various types of brain injury (Hao et al., 2017). Double immunofluorescence staining for ionized calcium-binding adapter molecule 1 (Iba1) and DAPI revealed that, in the Sham group, most Iba1-positive (Iba1^+^) microglia in the cortex were in a quiescent state, which was characterized by small cell bodies and long, thin, well-branched processes (Hao et al., 2017).

**Figure 4:**
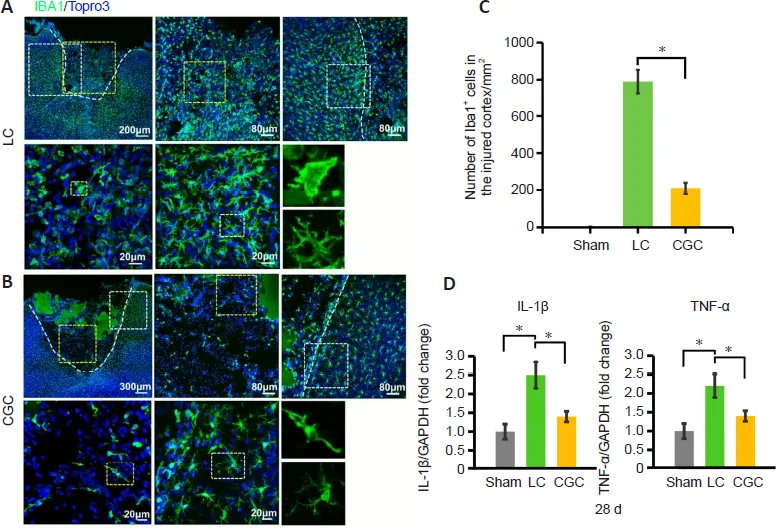
CGC suppresses the inflammatory response following TBCI. (A) On day 28 post-TBCI, microglia in the cortical lesion area exhibited pronounced morphological changes, with most Iba1^+^ cells showing an activated phenotype characterized by swollen cell bodies and shortened, thickened processes. Scale bars: 200, 80, and 20 μm. (B) CGC implantation markedly reduced the number of activated microglia in the lesion area, with most cells remaining in a quiescent state. These microglia showed small, flat cell bodies, slender processes, and clearly defined branches. Scale bars: 300, 80, and 20 μm. (C) Quantitative analysis of microglial numbers in the cortical lesion area of each group. (D) Quantitative analysis of the changes in the RNA expression levels of IL-1β and TNF-α in each group 28 days after the operation (*n* = 4). Data are presented as the mean ± SEM (one-way analysis of variance followed by Tukey’s *post hoc* test). *P < 0.05, *vs*. LC group. BrdU: Bromodeoxyuridine; CGC: chitosan–gelatin–collagen gel; GAPDH: glyceraldehyde-3-phosphate dehydrogenase; Iba1: ionized calcium-binding adapter molecule 1; IL-1β: interleukin-1 beta; LC: lesion control; TBCI: traumatic brain cortical injury; TNF-α: tumor necrosis factor-alpha.

As shown in **Figure 4A–D**, after TBCI, microglia in the peri-lesional cortex underwent pronounced morphological changes, with most Iba1^+^ cells showing an activated phenotype characterized by swollen cell bodies, shortened and thickened processes, and an amoeboid appearance (**Figure 4A** and **C**). CGC implantation markedly reduced the number of activated microglia and improved their morphology, shifting cells from an activated into a quiescent state (**Figure 4B** and **C**). Although CGC implantation could reduce the number of Iba1^+^ microglia in the brain cortical injury area after TBCI, what changes occurred in the gene expression levels of pro-inflammatory factors such as interleukin (IL)-1β and tumor necrosis factor (TNF)-α? The results of reverse transcription (RT)-polymerase chain reaction (PCR) analyses performed at 28 days post-operation suggested that the gene expression levels of the pro-inflammatory factors IL-1β and TNF-α increased after TBCI, and the implantation of CGC significantly reduced the expression of these two factors. These results suggested that CGC has some anti-inflammatory effects in the process of brain cortical injury repair.

#### Promotion of angiogenesis in the lesion area

**Figure 5:**
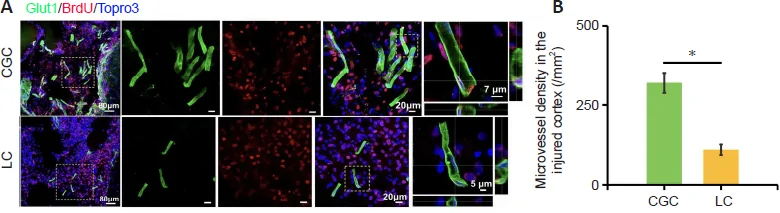
Regeneration of blood vessels in the cortical lesion area on postoperative day 56. (A) Numerous Glut1^+^/BrdU^+^ newborn vascular structures were observed within the cortical lesion area of the CGC group. In the LC group, no Glut1^+^/BrdU^+^ newborn vascular structures were detected, and only a very small number of Glut1^+^/BrdU^–^ vessels were present. Scale bars: 80, 20, 7, and 5 μm. (B) Quantitative analysis of vascular density in the cortical lesion area of each group. Data are presented as the mean ± SEM (Student’s *t*-test). *P < 0.05. BrdU: Bromodeoxyuridine; CGC: chitosan–gelatin–collagen gel; LC: lesion control.

The occurrence of angiogenesis is a critical factor influencing brain injury repair. Therefore, using Glut-1 as the marker for vascular endothelial cells, we next investigated the effect of CGC implantation on vascular regeneration following TBCI. On postoperative day 56, no Glut-1-positive (Glut-1^+^)/BrdU^+^ newborn vascular structures were detected in the cortical lesion area of the LC group (**Figure 5A**). Only a very small number of Glut-1^+^/BrdU-negative (BrdU^–^) vessels were observed. These may represent either residual vessels or vessels newly formed after postoperative day 7, warranting further investigation. In contrast, a large number of Glut-1^+^/BrdU^+^ newborn vascular structures were observed in the cortical lesion area of the CGC group. Laser confocal scanning confirmed the presence of typical vascular endothelial cells within the lesion area (**Figure 5A**). Quantitative analysis demonstrated that the density of regenerated blood vessels in the cortical lesion area of the CGC group was significantly higher than that in the LC group (*P* < 0.05; **Figure 5B**).

#### Inhibition of glial scar infiltration into the lesion area

After spinal cord injury, astrocytes proliferate extensively and migrate into the lesion area, forming both physical and chemical barriers that impede axonal regeneration (Li et al., 2009; Yang et al., 2015). Although scar formation limits the expansion of the injury volume caused by secondary damage, the scar itself does not promote axonal regeneration or functional recovery following spinal cord injury (Zhao et al., 2022).

**Figure 6:**
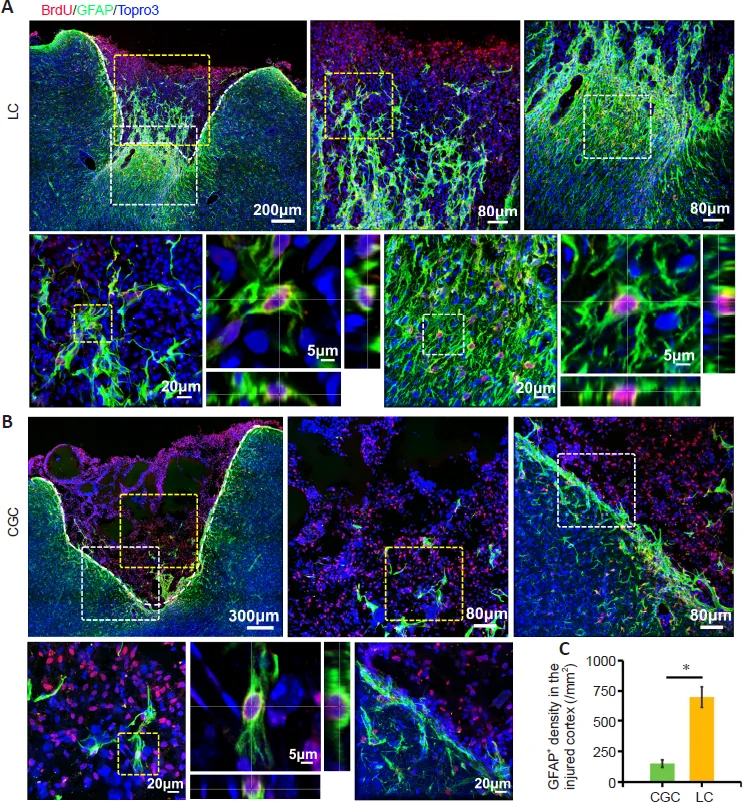
CGC gel implantation inhibits the infiltration of glial scars into the lesion area on post-TBCI day 56. (A) Numerous GFAP^+^/BrdU^+^ activated astrocytes with triangular morphology were observed in the lesion area (yellow box) and its boundary (white box) in the LC group. Scale bars: 200, 80, 20, and 5 μm. (B) Only a very small number of spindle-shaped GFAP^+^/BrdU^+^ activated astrocytes were observed in the lesion area (yellow box) and its boundary (white box) in the CGC group. Scale bars: 300, 80, 20, and 5 μm. (C) The quantitative analysis suggests that CGC implantation inhibited the infiltration of activated astrocytes into the lesion area. Data are presented as the mean ± SEM (Student’s *t*-test). *P < 0.05. BrdU: Bromodeoxyuridine; CGC: chitosan–gelatin–collagen gel; GFAP: glial fibrillary acidic protein; LC: lesion control; TBCI: traumatic brain cortical injury.

On day 56 post-TBCI, multiplex immunofluorescence staining for glial fibrillary acidic protein (GFAP), BrdU, and DAPI revealed numerous GFAP/BrdU double-positive (GFAP^+^/BrdU^+^) activated astrocytes with triangular morphology in the lesion area (yellow box) and its boundary (white box) in the LC group (**Figure 6**). In contrast, only a very small number of spindle-shaped GFAP^+^/BrdU^+^ activated astrocytes were observed in the lesion area (yellow box) and its boundary (white box) in the CGC group (**Figure 6**). The results of the quantitative analysis suggest that CGC implantation inhibited the infiltration of activated astrocytes into the lesion area (*P* < 0.05).

These results suggest that CGC implantation following TBCI could suppress inflammatory responses, reduce glial scar infiltration, and promote angiogenesis, thereby creating a favorable microenvironment in the brain lesion area that supports neural repair as well as the generation, maturation, and long-term survival of newborn neurons.

### Chitosan–gelatin–collagen gel promotes recovery of right forelimb sensorimotor function after traumatic brain cortical injury

**Figure 7:**
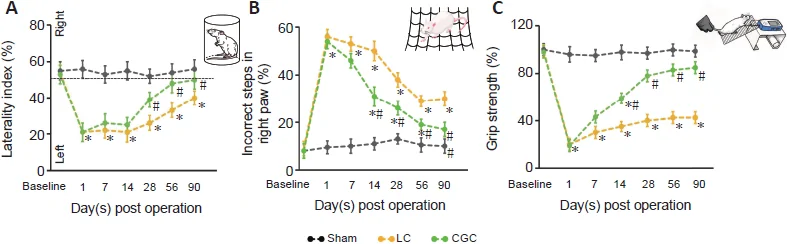
CGC promotes behavioral functional recovery after TBCI. (A–C) The performance of different groups in the cylinder test (A), grid-walking test (B), and grip-strength test (C) before and after TBCI. Data are presented as the mean ± SEM (*n* = 5, two-way analysis of variance). *P < 0.05 *vs*. Sham group; #*P* < 0.05, *vs*. CGCm-Cherry group. CGC: Chitosan–gelatin–collagen gel; LC: lesion control; TBCI: traumatic brain cortical injury.

The motor cortex is essential for skilled movements (Gonzalez et al., 2004). Thus, damage to the forelimb motor representation area following cortical injury typically results in impaired forelimb motor function in rats. To determine whether the newly generated brain tissue could restore forelimb motor function, we evaluated forelimb motor coordination and muscle strength using the cylinder test, grid-walking test, and grip-strength test (**Figure 7**).

After TBCI, rats showed decreased right forelimb wall touches during spontaneous exploration, increased right forepaw error rate, and reduced right forelimb grip strength (*P* < 0.05; **Figure 7A–C**), confirming successful establishment of the cortical injury model and demonstrating the forelimb motor dysfunction and muscle weakness induced by TBCI. In the cylinder test, assessment of forelimb preference (%) indicated that sham rats used their right forepaw to support their body weight in over 50% of spontaneous vertical explorations. However, after motor cortex injury, the frequency of right forepaw weight support decreased significantly below baseline levels. Over time, the LC group showed some spontaneous recovery of right forepaw use, but even by day 90 post-TBCI, performance remained below the normal baseline. In contrast, the CGC group exhibited significantly greater right forepaw use than the LC group on day 28 post-TBCI, although it still remained below baseline. By postoperative day 56, right forepaw use in the CGC group nearly returned to baseline and remained stable through 90 days (*P* < 0.05; **Figure 7A**).

In the grid-walking test, baseline error rates for the right forepaw were below 20% across all groups. Following motor cortex injury, the right forepaw error rate increased significantly. From day 14 to 56 post-TBCI, error rates in both the LC and CGC groups declined but remained significantly above baseline. By day 90, the right forepaw error rate in the CGC group had largely returned to baseline levels (*P* < 0.05; **Figure 7B**).

In the grip-strength test, rats grasped the metal grid with their right forepaw while their tails were gently pulled backward until the peak resistance force (g) was recorded. Motor cortex injury caused a marked reduction in the right forelimb strength. Although the LC group showed some spontaneous recovery over time, the right forelimb strength in this group remained below baseline on days 56 and 90. The CGC group showed partial recovery by day 28 and nearly full recovery to baseline strength by postoperative day 56 (*P* < 0.05; **Figure 7C**).

Together, these results suggest that CGC implantation increased right forepaw weight-bearing frequency, significantly reduced forepaw error rates during spontaneous exploration, and restored right forelimb strength to levels comparable with sham controls, thereby substantially improving sensorimotor deficits in the rat forelimb after TBCI.

### Relationship between newborn neurons and behavioral functional recovery revealed by viral ablation technology

**Figure 8:**
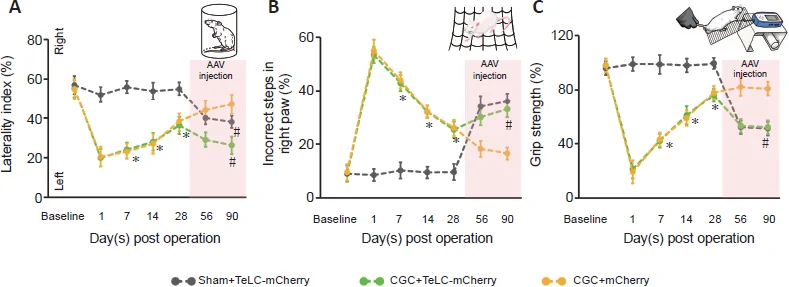
TeLC virus silences synaptic function and partially attenuates CGC-induced behavioral recovery. (A–C) The results of the cylinder test (A), grid-walking test (B), and grip-strength test (C). Behavioral data across multiple groups were analyzed using two-way repeated measures analysis of variance. Data are presented as the mean ± SEM (*n* = 5, two-way analysis of variance). *P < 0.05, *vs.* Sham + TeLC-mCherry group; #*P* < 0.05 *vs.* CGCm-Cherry group. AAV: Adeno-associated virus; CGC: chitosan–gelatin–collagen gel; TeLC: tetanus toxin light chain.

The results described above suggest that the CGC gel promoted the generation of newborn neurons in the cortical lesion area and facilitated behavioral recovery. To determine whether this functional recovery depended on the newborn neurons in the cortical lesion area, TeLC was injected into the cortical lesion area to ablate the synaptic function of these newborn neurons. On day 35 post-TBCI, rAAV-hSyn-TeLC-P2A-mCherry was injected into the cortical lesion area of rats in each group to specifically target synaptic activity. rAAV-hSyn-mCherry, expressing mCherry alone, served as the control virus. Behavioral analyses, including the cylinder test, grid-walking test, and grip-strength test, were performed to evaluate the right forelimb behavioral function under different intervention conditions after TBCI (*P* < 0.05; **Figure 8**).

Three weeks after injection, TeLC impaired right forelimb sensorimotor function in both the sham and CGC groups, as evidenced by deficits in the cylinder, grid-walking, and grip-strength tests. These results indicate that TeLC partially blocked normal cortical function in the Sham group and disrupted the CGC-induced behavioral recovery after TBCI. In contrast, rats in the CGC gel group injected with the control virus rAAV-hSyn-mCherry showed no impairment in right forelimb behavioral function and exhibited gradual, sustained improvement. These results suggest that the CGC gel-mediated improvements in forelimb behavioral function after TBCI are at least partly dependent on newborn neurons and their synaptic connections within the cortical lesion area.

## Discussion

TBCI directly damages cranial structures, affecting neurons, glial cells, and blood vessels in the injured brain regions, thereby leading to neurological dysfunction. Studies have shown that some degree of spontaneous functional recovery occurs following TBCI. This recovery is likely attributable to structural and functional compensatory changes within the damaged brain, which are mediated by mechanisms collectively known as neuroplasticity. However, in cases of severe brain injury, the limited capacity for compensation or neuroplasticity is insufficient to restore functional deficits in patients (Safdar et al., 2023).

To further enhance brain functional recovery after TBCI, researchers have explored various neuroprotective and regenerative strategies. These include transplantation of exogenous stem cells to replace neurons lost due to injury as well as the delivery of trophic factors to improve the local microenvironment, thereby promoting neuroprotection and regeneration. Nonetheless, transplantation of exogenous stem cells is associated with challenges such as immune rejection, limited cell sources, uncontrolled differentiation, and tumorigenic risk. Consequently, strategies that stimulate endogenous neural regeneration have become a focus of current research (Hao et al., 2024).

After brain injury, most quiescent microglia undergo morphological transformation into an intermediate activated state, which is characterized by swollen cell bodies and thickened, shortened, and widened processes (Hao et al., 2017). However, CGC implantation reduces the number of Iba1^+^ microglia in the cortical lesion area and shifts their phenotype from an activated state back toward quiescence. These findings suggest that resident microglia respond to TBCI and that CGC implantation can partially suppress microglial activation.

Another important histopathological change after brain trauma is damage to the cerebrovascular system. Both patients with TBCI and experimental animals exhibit alterations in cerebral vasculature (Arunkumar et al., 2019), including blood–brain barrier disruption, endothelial cell injury, and cerebrovascular hemodynamic disturbances. Therefore, improving cerebral circulation is critical for the prognosis of TBCI (Arunkumar et al., 2019). The rapid angiogenesis mediated by endothelial cells is essential for the survival of newborn neurons. After cerebral ischemia in rats, neurogenesis occurs concomitantly with angiogenesis, with the newly formed vascular network serving as a scaffold to facilitate the migration of newborn neurons (Liu et al., 2014). Our experiments suggested that CGC implantation promotes angiogenesis in the cortical lesion area, providing theoretical support for modulating the local injury microenvironment and enhancing the long-term survival of damaged brain tissue. Previous studies have confirmed the existence of endogenous NSPCs in the brain (Kempermann et al., 2018; Tobin et al., 2019; Babcock et al., 2021; Rodríguez-Barrera et al., 2021; Yang et al., 2021; Dumitru et al., 2025). After CNS injury, these NSPCs can be activated and differentiate into neurons and glial cells under specific environmental conditions (Yang et al., 2021; Hao et al., 2024). However, following TBCI, the local microenvironment in the cortical lesion area and surrounding brain tissue is typically characterized by inflammation, edema, and hypoxia. Such adverse conditions inhibit the differentiation of NSPCs into neurons, representing a major barrier to neural tissue regeneration. Therefore, improving the inhibitory microenvironment in the lesion area is critical for reducing neuronal death, enhancing the survival of newborn neurons, and promoting neural regeneration (Alves, 2014).

This study suggests that the CGC gel can improve the microenvironment of the non-neurogenic cortical lesion, shifting it from an inflammatory and ischemic state to one that is anti-inflammatory, neuroprotective, and pro-angiogenic. This creates a favorable “soil” for brain protection, regeneration, and functional recovery, enabling activated NSPCs that migrate to the cortical lesion area to differentiate into neurons and ultimately facilitating functional restoration.

## Limitations

IP transplantation of biological materials offers many advantages, such as the fact that no biological materials need to pass through the blood–brain barrier; consequently, this delivery system ensures transportation of the biological materials to the stroke cavity and overcomes potential off-target issues (Huang, et al., 2018; Bolan, et al., 2019). However, some limitations in the current clinical application of biological materials remain unresolved. First, the transplantation of biological materials in clinical practice still relies on implantation after craniotomy surgery, which significantly increases the risk of infection for patients. Second, ensuring that the transplanted biological materials remain confined to the injured area and do not change due to the flow of cerebrospinal fluid remains an unresolved issue. Finally, research on the use of biological materials for treating extensive brain injuries requires further improvement. Importantly, some recent clinical trials in humans and preclinical stroke studies in non-human primates have demonstrated that IP delivery appears to be a relatively safe and feasible administration route that is deserving of further clinical study (Chen, et al., 2013; Kalladka, et al., 2016; Cook, et al., 2017).

## Conclusions

In summary, CGC implantation promotes angiogenesis and inhibits both inflammatory responses and scar infiltration in the injured cerebral cortex, thereby creating a favorable “soil” (microenvironment) that supports the activation, migration, and neuronal differentiation of “seed” cells (endogenous NSCs). This study offers new insights into neuroprotection and regeneration following traumatic brain injury and provides guidance for future strategies combining neuroregenerative biomaterials with additional factors and/or exogenous NSCs for CNS repair. It also provides a theoretical foundation for the clinical translation of biomaterial hydrogels in treating CNS injuries.

## Additional files:

***Additional Table 1:*** *Information regarding primary and secondary antibodies.*

***Additional Figure 1:*** *FTIR absorption spectrum of the chitosan–gelatin–collagen (CGC) gel.*

Additional Figure 1 FTIR absorption spectrum of the chitosan–gelatin–collagen (CGC) gel.
The CGC gel exhibited two characteristic infrared absorption peaks: the O–H and N–H stretching vibration peak at 3435 cm^⁻1^, and the amide I band (C=O stretching vibration in the amide bond) at 1641 cm^⁻1^.

***Additional Figure 2:*** *The chitosan–gelatin–collagen (CGC) gel exhibits elastomeric characteristics.*

Additional Figure 2 The chitosan–gelatin–collagen (CGC) gel exhibits elastomeric characteristics.
Its viscosity remained relatively stable initially, and over time, both the viscosity and storage modulus reached steady values. The plateau viscosity was 5660 Pa·s at *t* = 200 s.

## Data Availability

*All relevant data are within the paper and its Additional files.*
